# Association Between Parents’ Self-Perceived Oral Health Knowledge and the Presence of Dental Caries in Their Children

**DOI:** 10.3390/clinpract15110204

**Published:** 2025-11-05

**Authors:** Andrea Coello Hidalgo, Ana Alvear Miquilena, Esteven Tipan Venegas, Yeslith Sandoval Sánchez, Diego Quiguango Farias, Maria Rodriguez Tates, Byron Velasquez Ron

**Affiliations:** 1 Carrera de Odontología, Universidad de Las Américas (UDLA), Quito 170516, Ecuador; andrea.coello@udla.edu.ec (A.C.H.); ana.alvear@udla.edu.ec (A.A.M.); e.tipanv@gmail.com (E.T.V.); yeslith-sandoval@hotmail.com (Y.S.S.); gaby.rodriguezt1@gmail.com (M.R.T.); 2 Carrera Ciencias de la Salud, Universidad de Las Américas (UDLA), Quito 170516, Ecuador; diego.quiguango@udla.edu.ec; 3 Carrera de Odontología, Department Prosthesis Research, Universidad de Las Américas (UDLA), Quito 170516, Ecuador

**Keywords:** oral health, parental knowledge, dental caries, self-assessment, schoolchildren, Ecuador

## Abstract

**Introduction:** Oral health in children is essential for their overall well-being, influencing nutrition, language development, and self-esteem. Dental caries represent one of the most prevalent chronic diseases in childhood. **Objective:** The objective of this study is to evaluate the association between parents’ self-perceived knowledge of oral health and the presence of dental caries in their children. **Materials and Methods:** A cross-sectional observational study was conducted with 1052 children aged 4 to 14 years and their parents in Quito, Ecuador. Parents completed validated questionnaires (OHIP-14, OIDP, CPQ, and OHQoL-UK) to assess their self-perceived oral health knowledge. Clinical examinations were performed to detect cavitated carious lesions. Statistical analysis included Chi-square tests and odds ratio (OR) calculations. **Results:** A significant association was found between lower parental knowledge and higher prevalence of dental caries in children (Chi-square = 16.245, *p* = 0.0062; OR = 18.18, 95% CI [1.80–183.75]). Most caries cases were found in children whose parents rated their knowledge as “good” or “very good,” suggesting a gap between perceived and actual knowledge. **Conclusions:** The findings highlight the need for targeted educational strategies that address both knowledge and behavioral practices in oral health, especially among parents with low self-perceived knowledge.

## 1. Introduction

Children’s oral health is an essential component of overall well-being, directly influencing nutrition, language development, self-esteem, and school performance. Dental caries, considered a multifactorial disease, represent one of the most prevalent chronic pathologies in childhood worldwide [1]. In Latin America, and particularly in Ecuador, studies on the influence of parents on their children’s oral health are scarce, which limits the design of effective educational strategies.

Children’s oral health plays a vital role in their overall development, affecting nutrition, communication, and self-esteem. Dental caries remain one of the most common chronic conditions in childhood, with multifactorial origins including diet, hygiene habits, and parental influence [2].

A number of factors contribute to the development of cavities, including hygiene habits, sugar consumption, access to dental services, and caregivers’ level of knowledge. The scientific literature has shown that parental knowledge about oral health is significantly associated with the prevalence of caries in children [3]. However, this knowledge does not always translate into proper practices, suggesting a gap between perception and action.

From a theoretical perspective, the Health Belief Model [4] states that the perception of risk, expected benefits, and perceived barriers influence the adoption of healthy behaviors [5]. In this context, parents may overestimate their knowledge without implementing effective prevention measures, such as supervised brushing or the proper use of fluoride [6,7].

In Ecuador, initiatives such as World Oral Health Day have sought to promote healthy habits, but inequalities in access to information and services still persist, especially in rural areas. This study is part of a community project in vulnerable areas of northwestern Quito, where the association between parents’ self-perceived knowledge and the presence of caries in their children was evaluated. Research hypothesis: The lower the parents’ self-perceived knowledge about oral health, the higher the prevalence of caries in their children [8].

The oral health of children is critical to their overall well-being, and it influences aspects such as nutrition, language development, and self-esteem. Dental caries represent one of the most common chronic diseases that occur in childhood, affecting a large proportion of the world’s pediatric population. A number of factors contribute to the occurrence of dental caries, including oral hygiene habits and diet. However, the role of parents or guardians in the establishment and maintenance of healthy habits is crucial for the prevention of this pathology. Studies have shown a significant association between parents’ level of knowledge about oral health and the prevalence of cavities in their children [9]. There is a statistically significant relationship between the frequency of dental caries in schoolchildren and their mothers’ degree of oral health knowledge. The frequency of dental caries in children was 92.9%, while the mother’s degree of knowledge about oral health was distributed as follows: high (32.7%), medium (43.4%), and low (23.9%) [10]. Children whose parents had a low level of knowledge about oral health had a higher prevalence of caries compared to those whose parents had a high level of knowledge. These findings highlight the importance of not only educating parents but also ensuring that this knowledge translates into proper oral hygiene practices at home [11]. Oral health education from an early age is essential. Initiatives such as World Oral Health Day celebrations aim to promote healthy habits and raise awareness among the population about the importance of dental care [12]. In addition, community programs that teach brushing techniques to children and their families have been shown to be effective in improving oral hygiene. Notably, although parents may have adequate knowledge about oral health, factors such as socioeconomic status, access to dental services, and cultural practices can influence the prevalence of caries in children [13]. Therefore, it is essential to address the oral health of children from a comprehensive perspective that considers parental education and socioeconomic conditions as well as access to health services. The research hypothesis proposed that the lower the degree of oral health knowledge of parents, the greater the prevalence of caries in their children [14]. Parental knowledge about oral health is a key determinant in the prevention of caries. Studies have shown that children whose caregivers possess limited oral health knowledge are more likely to develop caries. However, self-perceived knowledge may not always reflect actual understanding or effective practices [15].

Despite global efforts to promote oral health, gaps persist in the translation of knowledge into behavior, particularly in socioeconomically vulnerable populations. In Ecuador, local data on parental influence in oral health are scarce, making this study relevant for public health planning [16]. The research hypothesis is that parents with lower self-perceived oral health knowledge will have children with a higher prevalence of dental caries [17]. The alternative hypothesis is that parents with higher self-perceived oral health knowledge will have children with a lower prevalence of dental caries. The objective of this study was to analyze the association between parents’ self-perceived oral health knowledge and the prevalence of dental caries in their children.

## 2. Material and Methods

The inclusion criteria were as follows:

- Parents (male or female) with at least two children.
- Children between 4 and 14 years of age.
- Signed informed consent and assent of the minors and their legal guardian.
- Children had to be in good general health, without chronic systemic conditions.
- Participants with medical conditions that could affect oral health (e.g., congenital anomalies, and xerostomia) were excluded [18,19,20].

## 3. Statistical Analysis

The data were analyzed with the R software version 4.3.1 and its RStudio IDE 2025.09.1+401 environment [21,22]. Descriptive and inferential tools were applied, including the following:

- Chi-square independence test.
- Odds ratio (OR) calculation with 95% confidence interval.

The variables analyzed were qualitative and did not meet normal assumptions, so non-parametric tests were used [23,24].

## 4. Results

A total of 1052 children participated in the study, with a predominance of females (64.41%). The age distribution was as follows: 35.68% were between 4 and 8 years, 30.92% were between 8 and 12 years, and 33.40% were between 12 and 14 years. Regarding dental caries, 98.29% of children were free of cavitated lesions, while 1.71% presented with caries. (Table 1) The clinical examination focused exclusively on cavitated lesions, excluding white spot lesions and early demineralization. Parents’ self-perceived knowledge about oral health was categorized as follows:

- Good: 68.8%
- Very good: 20.8%
- Excellent: 0.19%
- Fair: 8.75%
- Not knowledgeable: 1.05%
- Terrible: 0.38%

**Figure 1:**
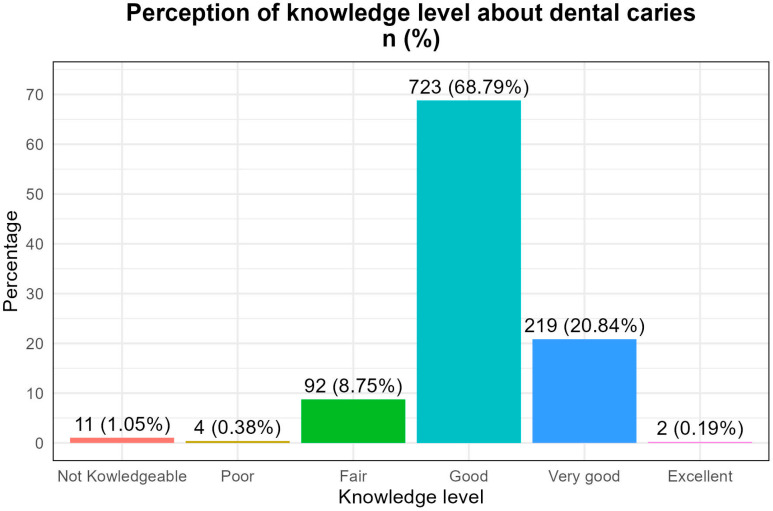
Perception of the level of knowledge of the legal representatives of minors.

The distribution of caries cases showed the following:

- A total of 61.1% of children with caries had parents who rated their knowledge as “good”.
- A total of 33.3% had parents who rated it as “very good”.
- Only one case was found among parents who rated their knowledge as “terrible”. (Figure 1)

A Chi-square test revealed a statistically significant association between parental knowledge and caries presence (χ^2^ = 16.245, df = 5, *p* = 0.0062).

The odds ratio (OR) for caries in children of parents with low knowledge was 18.18 (95% CI [1.80–183.75]), indicating a substantially increased risk (Figure 1).

To determine if there is a statistically significant association between the presence of caries in children and the level of knowledge perceived by their parents or representatives about oral health, a Chi-square test of independence was applied. The result obtained was a Chi-square statistic of 16.245 with five degrees of freedom and a *p* value of 0.0062. Given that the *p* value was lower than the conventional significance threshold of 0.05, the null hypothesis of independence between the variables was rejected, which indicated that there is a significant association between the level of perceived knowledge and the presence of dental caries.

This finding supports the hypothesis proposed in this study: the lower the parents’ knowledge of oral health, the higher the prevalence of caries in their children. Further analysis should consider potential confounding factors.

The statistical significance obtained reinforces the hypothesis put forward in this study: the lower the parents’ knowledge of oral health is, the higher the prevalence of caries in their children. However, although the Chi-square test confirms the existence of a relationship, it does not allow causality to be established. In addition, the analysis should be complemented with the evaluation of possible confounding factors, such as socioeconomic status, the formal education of parents, access to dental services, and the actual implementation of oral hygiene habits at home [25] (Table 2).

**Figure 2:**
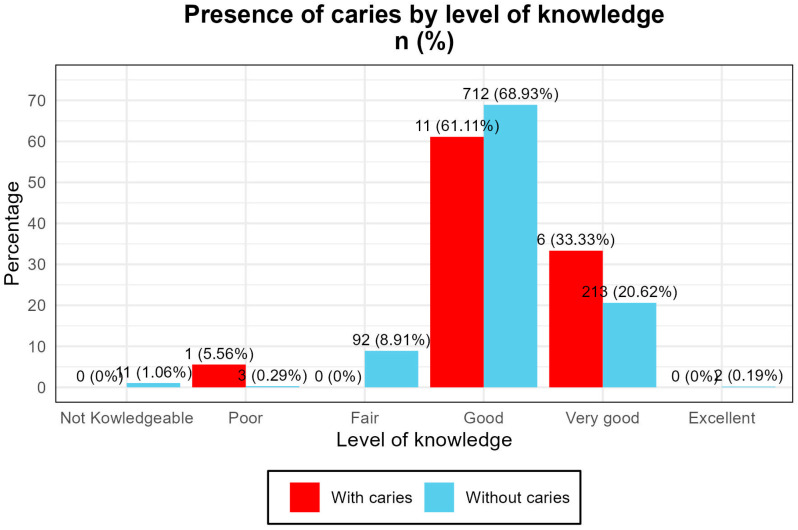
Presence of caries in children group according to the level of knowledge of their representatives.

The inclusion of educational programs aimed at parents and guardians, as well as community campaigns aimed at improving practical knowledge about oral hygiene, could significantly contribute to reducing the incidence of caries in the child population, as supported by successful initiatives described in the literature. [26] (Figure 2).

To quantify the strength of the association between low oral health knowledge among parents or guardians and the presence of dental caries in their children, the odds ratio (OR) was calculated. The analysis revealed an OR of 18.18, with a 95% confidence interval [1.80, 183.75]. This result suggests that children whose parents have a low level of knowledge are approximately 18 times more likely to develop cavities compared to those whose parents have higher levels of knowledge.

The confidence interval does not include the null value (OR = 1), confirming that this association is statistically significant. (Table 3) These findings are consistent with the scientific literature, where it has been observed that deficient knowledge of oral health among caregivers significantly increases the risk of caries in childhood. This relationship may occur due to the lower probability of implementing proper brushing habits, the lack of knowledge about the correct use of fluoride, or the lack of criteria to identify early signs of oral disease.

Importantly, this type of relationship has also been documented in contexts of socioeconomic vulnerability, where oral health knowledge is compromised by multiple barriers, including access to health services, parental education, and cultural beliefs. Therefore, beyond information campaigns, a multidimensional approach that combines oral health education with community, school, and family interventions that promote the effective application of knowledge is needed [27].

The magnitude of the ORs obtained reinforces the need to design specific strategies targeting parents with a low level of knowledge, who represent a high-risk group in terms of children’s oral health. Thus, caries prevention should not be limited to clinical intervention but should be approached from an educational and social perspective that increases family empowerment.

## 5. Discussion

This study identified a significant association between parents’ self-perceived knowledge about oral health and the presence of caries in their children. Although most parents rated their knowledge as “good” or “very good,” cases of caries were more frequent in these groups, suggesting a discrepancy between perception and actual practice. Previous studies have documented that the subjective perception of knowledge does not always translate into adequate oral hygiene behaviors, especially in contexts of socioeconomic vulnerability. [28,29] This finding can be explained by the phenomenon of social desirability bias or overconfidence, where parents tend to overestimate their knowledge for cultural, educational, or social reasons [30].

The international literature supports this observation. Research has shown that the limited knowledge of caregivers is associated with a higher prevalence of caries in children [31]. In addition, the Health Belief Model suggests that perceived risk perception and perceived barriers influence the adoption of preventive behaviors, reinforcing the need to evaluate not only knowledge, but also its practical application [32].

This also highlights the importance of considering structural factors such as access to dental services, parents’ educational level, and cultural practices. In rural areas of Ecuador, these barriers can limit the implementation of healthy habits, even when theoretical knowledge exists.

The results of this study revealed a significant association between the level of oral health knowledge of parents and the prevalence of dental caries in children, which is consistent with previous research that was conducted in similar contexts [31]. This relationship supports the hypothesis that parental knowledge acts as a protective factor against the development of oral diseases in childhood. Although a majority of participants rated their knowledge as “good” or “very good”, the observed high prevalence of caries suggests that there is a gap between perceived knowledge and effective oral care practices [33].

Therefore, it is recommended that oral health promotion programs are not limited to the transmission of information, but include practical interventions and family accompaniment [34]; are culturally adapted and accessible, integrating into community spaces such as schools and health centers; and use validated instruments to assess real knowledge and daily practices.

Children whose parents demonstrated a low level of knowledge had a higher frequency of caries, which suggests that the understanding and application of appropriate oral hygiene practices at home protect against this pathology [34].

The cross-sectional design of the study allows the identification of associations, although it does not establish causality. However, these findings are consistent with previous research findings that highlight the importance of the family environment in the prevention of oral diseases [35]. The literature has shown that children whose parents have a higher level of education and knowledge related to oral health have a lower incidence of caries, which is consistent with the results of this research [36]. In terms of social inequalities, this pattern is even more accentuated in contexts with high economic and educational vulnerability. Research has documented how poverty, low-level education, and cultural barriers negatively influence access to oral health knowledge and services [37], perpetuating a cycle of dental disease that primarily affects at-risk pediatric populations. This finding is also corroborated by regional studies indicating that parental knowledge acts as a key social determinant for children’s oral health. Various studies have pointed out that the self-perceived knowledge does not always translate into proper oral hygiene behaviors. Parents may overestimate their understanding of oral health or, even with adequate knowledge, may face structural or cultural barriers that hinder its implementation. Factors such as limited access to dental services, lack of ongoing educational programs, and the influence of family habits may contribute to this discrepancy. In addition to basic knowledge, many parents, while recognizing the importance of daily brushing and regular visits to the dentist, do not apply these habits with their children consistently [38]. Studies support the idea that knowledge alone, without action, lacks impact. It is essential to adopt intervention strategies that combine clear information with family and community support mechanisms to guarantee practical implementation. The evidence suggests that traditional information campaigns have been insufficient to reduce the prevalence of caries, so a comprehensive approach that involves schools, health systems, and family life in homes is needed [39]. Programs that promote oral health must consider psychosocial, cultural, and structural factors to achieve real and sustained change. A relevant finding is that only a small percentage of participants rated their knowledge as “excellent”, indicating a clear opportunity for educational interventions. The implementation of oral health promotion programs targeting parents and caregivers could have a significant impact on reducing the occurrence of childhood caries. These programs must be culturally appropriate, accessible, and sustainable, integrating into spaces such as schools, health centers, and communities [39]. In addition, it is important to consider that the self-perceived knowledge can be influenced by subjective factors, such as personal confidence or previous experience with dental services [40]. As such, future research could complement self-perception questionnaires with objective assessments of knowledge and observations of actual oral hygiene practices at home. Finally, this study contributes to the evidence supporting the need for comprehensive public policies that include oral health education as an essential component. The promotion of healthy habits from childhood, with the informed support of parents, is key to improving the oral health of the child population and reducing health inequalities.

In addition, future research should incorporate longitudinal designs to assess the impact of educational interventions on parental behavior and children’s oral health. It would also be useful to supplement perception questionnaires with direct observations of hygiene habits in the home.

This study provides relevant evidence for the design of public policies aimed at promoting children’s oral health, underlining the need to strengthen the link between knowledge, attitude, and practice in the family environment.

## 6. Conclusions

This study confirms the existence of a significant relationship between the knowledge of parents or guardians and the presence of dental caries in their children. These findings reinforce the need to design more effective educational strategies aimed at both the transmission of information and the transformation of daily practices in the family environment. These findings also highlight the importance of addressing the structural inequalities that limit access to information and oral health services in vulnerable communities. It is recommended that public health policies integrate continuing oral education programs into schools and community centers, with an emphasis on parental involvement. Finally, applied research should be promoted to allow these strategies to be adapted to the sociocultural realities of each community to achieve a lasting impact on children’s oral health.

## 7. Limitations of the Study

- The cross-sectional design prevents causal inference.
- The reliance on self-perception may introduce bias.
- The exclusion of non-cavitated lesions may underestimate caries prevalence.

## 8. Implications for Public Health

- Educational strategies should target parents with low self-perceived knowledge.
- Programs must be culturally adapted and integrated into schools and communities.
- Future research should include objective assessments of knowledge and longitudinal designs to evaluate behavioral outcomes.

In conclusion, improving parental understanding and practices is essential for reducing childhood caries and promoting oral health equity.

## Figures and Tables

**Table 1:** Summary of information collected by the minors’ legal representatives.

Variable	Count	%	Accumulated%
Sex			
Man	374	35.59	35.59
Woman	677	64.41	100.00
Age			
to 4–8 years	375	35.68	35.68
to 8–12 years	325	30.92	66.60
to 12–14 years	351	33.40	100.00
Presence of caries			
With cavities	18	1.71	1.71
No cavities	1033	98.29	100.00

**Table 2:** Chi-square relationship test between the level of knowledge of the parents and the presence of caries in the children.

Relation	Statistical	df	*p* Value
Presence of caries vs. knowledge	16.245	5	0.0062

**Table 3:** Odds ratio between low knowledge of parents and guardians and the presence of caries in children.

Relation	Odds Ratio	IC95% [LI − LS]	*p* Value
Low knowledge and presence of caries	18.18	[1.80, 183.75]	0.014

## Data Availability

Data are contained within the article.

## Abbreviations

ORs	odds ratios
MSP	Health Public
OCP	heavy crude oil pipeline
OHIP-14	Oral Health Impact Profile
OIDP	Oral Impacts on Daily Performance
CPQ	Child Perceptions Questionnaire
OHQoL-UK	Oral Health-Related Quality of Life—UK
